# Imagining sustainable energy and mobility transitions: Valence, temporality, and radicalism in 38 visions of a low-carbon future

**DOI:** 10.1177/0306312720915283

**Published:** 2020-05-06

**Authors:** Benjamin K Sovacool, Noam Bergman, Debbie Hopkins, Kirsten EH Jenkins, Sabine Hielscher, Andreas Goldthau, Brent Brossmann

**Affiliations:** Science Policy Research Unit (SPRU), University of Sussex, UK; Center for Energy Technologies, Aarhus University, Denmark; Science Policy Research Unit (SPRU), University of Sussex, UK; School of Geography and the Environment, University of Oxford, UK; Science, Technology and Innovation Studies (STIS), University of Edinburgh, UK; Science Policy Research Unit (SPRU), University of Sussex, UK; Institute for Advanced Sustainability Studies and Willy Brandt School at the University of Erfurt, Germany; Tim Russert Department of Communication & Theatre, John Carroll University, USA

**Keywords:** energy discourse, futures, imaginaries, sociotechnical transitions, visions

## Abstract

Based on an extensive synthesis of semi-structured interviews, media content analysis, and reviews, this article conducts a qualitative meta-analysis of more than 560 sources of evidence to identify 38 visions associated with seven different low-carbon innovations – automated mobility, electric vehicles, smart meters, nuclear power, shale gas, hydrogen, and the fossil fuel divestment movement – playing a key role in current deliberations about mobility or low-carbon energy supply and use. From this material, it analyzes such visions based on rhetorical features such as common problems and functions, storylines, discursive struggles, and rhetorical effectiveness. It also analyzes visions based on typologies or degrees of valence (utopian vs. dystopian), temporality (proximal vs. distant), and radicalism (incremental vs. transformative). The article is motivated by the premise that tackling climate change via low-carbon energy systems (and practices) is one of the most significant challenges of the twenty-first century, and that effective decarbonization will require not only new energy technologies, but also new ways of understanding language, visions, and discursive politics surrounding emerging innovations and transitions.

## Introduction

Visions and narratives of energy futures have become a particularly powerful force in research, being present in the construction of energy and climate scenarios, forecasts, and policy analysis (O’Neill et al., 2017; Sovacool, 2019). Outside of the research community, business analysts, regulators, titans of industry and inventors (among others) continually devote a significant amount of effort towards developing and deploying futuristic narratives and images for political and economic ends. In the public domain, users, consumers, citizens and the media also frequently invent, modify, circulate and/or resist such narratives (Mason, 2006). Such visions serve symbolic but also instrumental ends, soliciting public, political, and even financial support for a variety of low-carbon technologies (Curran, 2012; Delina and Janetos, 2018; Fortes et al., 2015). Visions, fantasies and narratives therefore have relevance for all stakeholders who are concerned about energy and climate policy decisions, including innovation, technology choice and commercialization.

Notwithstanding their prominence and importance, little academic work has attempted to engage the topic of visions and fantasies empirically or theoretically in a systematic and comparative manner, while at the same time connecting them to pressing policy concerns such as low-carbon transitions. Our awareness of this research gap comes from the work of a five-year research center – CIED, the Centre on Innovation and Energy Demand – examining innovation and energy demand from a socio-technical and whole systems approach, in which most of the authors were involved, and where most of the case studies were conducted. Readers can look to CIED (2019) for an overview, to Geels et al. (2018) for CIED’s overall research strategy and to Jenkins and Hopkins (2019) for its flagship output, a book.

This article examines 38 distinct visions associated with seven different innovations – automated mobility, electric vehicles, smart meters, nuclear fission, shale gas, hydrogen, and the fossil fuel divestment movement – each of which plays a key role in current deliberations about future energy supply and use. The article examines:

1. *Rhetoric*, or the ideographs, narratives, symbolic cues and recurring phrases that support or weaken the discursive appeal of a particular low-carbon innovation,
2. *Agents*, or the problems, actors, characters and plotlines (including the technology being the hero, or nature or climate being the villain, for instance) involved in articulating such visions, and
3. *Strategies and contestations*, or how such visions and discourses interact with agents and each other, especially in response to external selection pressures and the evolution of competing narratives.

The article is motivated by the premise that tackling climate change via low-carbon energy systems (and practices) is one of the most significant challenges of the 21st century, and that success will require not only new energy technologies and other innovations, but also new ways of understanding language, visions and discursive politics that define new futures. Sovacool and Brossmann (2013: 211) have noted that *imaginings* of energy technologies play an important role in decisions made about such technologies, presenting a ‘critical social facet of energy transitions’. Cozen et al. (2018: 289) write: ‘A sustainable future depends on how we think about, *communicate about*, and use energy’ (emphasis added). This paper therefore both identifies a collection of novel emerging visions and seeks to advance our conceptual understanding of how such visions differ in terms of their rhetorical features, valence, temporality, and radicalism.

The article’s methodology is based on an extensive synthesis of evidence and analysis including semi-structured interviews, media content analysis, and systematic reviews. The article is inherently cross-technological in its examination of visions, looking at low-carbon technologies across the domains of electricity supply, transport and mobility, industry, and household energy use. It is spatially and temporally comparative, examining several specific geographic contexts where such visions and narratives play out: nationally in the United Kingdom, regionally in Eastern Europe, and globally in the epistemic communities connected to nuclear power, divestment, and automated mobility. Taken together, this allows us to consider a broad range of visions and narratives expressed in a variety of ways, each seeking to shape some aspect of our future energy landscape.

## Conceptual approach: Visions, fantasy, ideographs and cues

The core terms and concepts in the study – vision, fantasy, ideograph, and cue – interrelate. It is helpful to define each in turn, with a summary offered by Table 1.

**Table 1.** Key terms used in this study.

Term	Definition
Vision	A description of what could occur in the near-term, mid-term, or long-term future. While shaped by ideological constraints, visions reveal alternative narratives or futures, thus inviting contestation within themselves, and between alternative perspectives
Fantasy	A narrative that dramatizes a vision, making it salient to audiences through dramatic devices and/or recurring themes
Ideograph	A term of cultural and political collective commitment that embraces historical norms sufficiently to guide subsequent discourse.
Cues	Key words or phrases that resonate symbolically with particular audiences.

*Visions* deploy stories, narratives, or scenarios that reveal fundamental patterns of human reasoning, and how humans communicate their thinking to others, in a future oriented context. Berkout (2006) argues that visions can play at least five different, important and active roles. They can:

- map a possibility space by identifying a realm of plausible alternates and the means for reaching them,
- offer a heuristic device for revealing the specific problems that need to be resolved in order for a vision to be realized,
- enable the identification of stable frames for target setting and monitoring progress,
- specify metaphors and relevant symbols, narratives, or moralities that bind together different stakeholder groups, or
- bring together capital, knowledge, networks, skills, and other resources so that action can be coordinated and focused.

Such articulated visions are simultaneously rational and allegorical, reflecting the ability to story-tell and construct myths as much as to reason, and showing that reality is symbolically mediated (Brown et al., 2000; Miller et al., 2015; Noppers et al., 2014).

Our use of the term *fantasy* is more precise: it refers to ‘a storyline that captures the human need to experience and interpret drama’. For Bormann (1972), ‘fantasy’ refers to the way that communities of people share their social reality, a creative interpretation of events that fulfill a psychological and rhetorical need. It is not to be mistaken for something that is necessarily imaginary or pejorative. Fantasies often have ‘symbolic cues,’ recurring phrases, terms, or slogans such as ‘my precious.’ In contrast to Bormann, we focus almost exclusively on the narrative dimension of fantasy. As a guiding lens, we rely on a particular type of fantasy, one of utopianism and dystopianism. Michael (2000: 23) notes:

Representations of the future are from the outset engaged in a sort of pre-emptive argumentation over whether the projected state of affairs leads to good or bad … Representations of a good future can be charged with ‘talking up’ the future - that is, the enunciation of a particularly positive future can generate ‘optimism’ (or bullishness in markets). Similarly, a slight hint of negativity regarding the future can precipitate panic.

Utopian visions of technology have been explored in the academic literature more than dystopian visions. Berkout (2006: 302) writes: ‘[U]topias represent examples of radical and more fully worked visions of the future. Their aim is to break the bonds of the existing order, to exemplify an alternative order and to inform collective action in pursuit of that order.’

Yet Segal (1994, 2005) offers an alternative view of ‘technological utopia’ as a mode of thought that promotes technology as bringing about a perfect society. For instance, Eames et al. (2006) show how visions of a hydrogen economy touch upon six overarching utopian themes such as:

- ending dependence on insecure supplies of energy,
- decentralizing energy via community ownership of energy systems and smaller and more distributed sources of supply,
- fundamentally reforming social values towards sustainability,
- allowing humanity to retain its current lifestyles,
- harnessing technical progress, knowledge, and innovation, and
- creating employment and staying in the international race for economic competitiveness.

The utopian elements of technological fantasies have therefore led proponents and sponsors to exaggerate potential benefits and downplay risks of many different technologies (Corn, 1986; Sturken et al., 2004). Marvin (1988) warns that technological utopianism can also promote a ‘cognitive imperialism’ where social and political relations become reduced and technologically determined. Hornsey and Fielding (2016) analyzed reactions to different messages addressing global warming, and found that optimistic or utopian messages reduce the sense of risk from global warming, and its associated distress, and are less successful in motivating action than pessimistic messages.

Utopian and dystopian fantasies shaped by *ideographs*, powerfully recurring themes or ideologies behind visions. McGee (1980) originally developed the concept of an ideograph to explain how language, or rhetoric, can reinforce or linguistically implement ideology, or power, by manipulating mass consciousness. According to him, human beings are ‘conditioned’ to a vocabulary of concepts that function as guides, warrants, reasons or excuses for behavior and belief. An ideograph is defined (McGee, 1980: 15) as ‘a high order abstraction, representing collective commitment to a particular but equivocal and ill-defined normative goal. It warrants the use of power, excuses behavior and belief which might otherwise be perceived as eccentric or antisocial, and guides behavior and belief into channels easily recognized by a community as acceptable and laudable.’ McGee suggests that ideology is in practice a political language composed of ideographs that signify a collective commitment. Andsager (2000: 578) sees ideographs as ‘special words or phrases that express public values’, and Berkout (2006: 307) understands them as a ‘set of guiding concepts representing unifying ideas and ideals’. Examples include deploying the words ‘natural’ and ‘sustainable’ as they relate to products and practices, with all of the social context or judgments those words imbue.

Ideographs about technology signify a baseline of public and political commentary, and often relate to common rhetorical tropes such as ‘freedom’, ‘quality’, ‘prosperity’, and ‘safety’. Van Lente (2000) muses that perhaps the most prominent ideograph connected to technology has been that of ‘continual progress’, an idea reaching as far back as the Enlightenment in the 1700s. Liao (2012) similarly identifies ideographs connected to augmented reality technologies such as ‘education’ or ‘privacy violations’. In our analysis below, we extend this work and identify no fewer than fourteen ideographs: progress & innovation, efficiency, profit & economic growth, environmental sustainability, safety, liberty & autonomy, employment, education, privacy, duty & responsibility, resistance, national security, inevitability, and authoritarianism.

Since all words are symbols that provide meaning for a referent, the category of *cues* is clearly the broadest. Cues represent words or phrases that trigger contextual knowledge for people actively involved in a relevant conversation or vision.

Toward this end, our four concepts interrelate, as ideographs are necessarily cues, but they represent an extremely narrow set of terms whose meaning is grounded in the ideological assumptions of an audience. Ideographs are so rooted in a people’s collective belief system that they serve to as perceptual filters for everyone within a collective, regardless of power. An ideograph is a cue whose meaning is culturally ingrained; due to historical usage, its meaning is thought to be universally accepted and generally beyond challenge (although unspecified differences within cultures remain). Who can object to progress? Invoking ‘progress’, though, can strategically obscure important questions (progress for whom, in what way, at what cost?). Cues tend to work as perceptual blinders (Burke, 1945, 1950, 1966), prompting audiences to look at issues from a particular perspective and obscuring other views. Visions are also limited by ideological constraints, but they can embrace specific interpretations of our collective agreements in efforts to provide alternative views of potential futures. To make those visions more appealing to people who tend to embrace narrative decision-making processes, fantasies use drama to tether the visions to dramas and recurring themes with which people identify. These fantasies tend to use recurring themes or plots to articulate their stories.

Moreover, our examination of visions, fantasies, ideographs and cues yields similar insights to work that focuses on narratives and imaginaries, albeit with differing results and emphasis. A narrative, in its broadest sense, refers to a carefully crafted or mobilized story or an account of events (Miller, 2012). While all visions are thus a form of narratives, many narratives fall outside the scope of vision. Visions refer to a specific type of narrative focused only on a desirable or undesirable future. Imaginaries refer to ‘collectively held, institutionally stabilized, and publicly performed visions of desirable futures, animated by shared understandings of forms of social life and social order attainable through, and supportive of, advances in science and technology’ (Jasanoff, 2015: 4). The pioneering work of Jasanoff and Kim (2009, 2013) has revealed such imaginaries around the use of nuclear power in South Korea and the United States. However, our visions do not fit neatly within the imaginaries concept because they are often institutionally unstable, undesirable, and unsupportive of advances in science and technology. We thus view our approach to low-carbon visions as a complement to, rather than a substitute for, ongoing research on narratives and imaginaries.

## Research design: Selection of low-carbon innovations and qualitative meta-analysis

To explore the discursive dimensions of energy visions and fantasies, we relied on a comparative approach that examined seven different low-carbon innovations. To do so, we are building on our collective earlier work (see Table 2), although the synthetic and comparative analysis presented here is entirely new.

**Table 2.** Summary of technological case studies and methods for this meta-analysis.

Innovation	Method of data collection*	Country focus	Number of documents or statements collected (N)	More details in:
(a) Automated mobility	Content analysis of freight industry and mass media documents	Global	107	Hopkins and Schwanen, 2018a, 2018b
(b) Battery electric vehicles	Content analysis of major scenarios and forecasts about electric vehicles and low-carbon vehicles	United Kingdom	16	Bergman, 2017, 2018; Bergman et al., 2017
(c) Smart meters	Content analysis of broadsheet and tabloid newspaper articles	United Kingdom	205	Hielscher and Kivimaa, 2018; Hielscher and Sovacool, 2018
(d) Nuclear power	Research interviews	United Kingdom	36	-
(e) Shale gas fracking	Research interviews	Eastern Europe	74	Goldthau, 2018; Goldthau and Sovacool, 2016
(f) Hydrogen	Content analysis of project documents and public media (52) and interviews (62)	United States	114	Sovacool and Brossmann, 2010
(g) Divestment	Research interviews	Global	12	Bergman, 2018

* Refers to material collected through the duration of a five-year research effort known as the Centre on Innovation and Energy Demand.

We selected seven distinct low-carbon innovations covering different scales or sectors (households, industry, transport), services (electricity, mobility, freight), fuels (fossil, nuclear, renewable), and types of innovation (technical, social). Three of these deal with energy use and demand, or the ‘prime movers’ of energy or mobility services:

(a) Automated vehicles,
(b) Battery electric vehicles, and
(c) Smart meters.

Three of these deal with the supply or storage of low-carbon energy:

(d) Nuclear fission (electricity),
(e) Shale gas (electricity and heat), and
(f) Hydrogen (energy storage).

A final one deals with social innovation or grassroots activism:

(g) Fossil fuel divestment.

In choosing these innovations, and not others, we were working with empirical material we had collected previously in our research. That said, we also wanted to avoid low-carbon innovations that had already been analyzed for their visions and discourse, notably clean coal (Kuchler and Bridge, 2018; Marshall, 2016; Rafey and Sovacool, 2011), biofuel (Fatimah 2015; Kuchler, 2014; Levidow and Papaioannou, 2013), wind energy (Jepson et al., 2012; Karlsen, 2018; Korsnes, 2016), and solar energy (Cloke et al., 2017; Curran, 2012; Phillips and Dickie, 2014; Rosenbloom et al., 2016; Simmet, 2018). We also wanted to choose innovations seen as having significant potential to actually reduce carbon emissions within the next few decades. Automated vehicles and electric vehicles have been recently advanced as a means to dramatically decarbonize the transport sector and even ‘revolutionize’ mobility (Axsen and Sovacool, 2019; Sperling, 2018). Smart meters, nuclear reactors, shale gas and hydrogen all feature centrally in projections about low-carbon energy use undertaken by the International Energy Agency (2019a) and the European Commission’s (2017) Strategic Energy Technology Plan. The carbon footprint of shale gas production remains expressly subject to long-standing contestations (e.g. Cathles et al., 2012; Howarth et al., 2011). Nevertheless, unconventional energy has contributed to a surge in global LNG trade, and in some contexts globally available natural gas is considered an important ‘bridge fuel’ to a low-carbon future (IEA, 2019b). The December 2019 political agreement on an EU-wide classification system for sustainable investments in principle also recognizes nuclear power as a transitional fuel (making it, however, contingent to a set of ‘do no significant harm criteria’) (European Commission, 2019). Fossil fuel divestment has been heralded as ‘particularly pertinent’ to financing the global low-carbon energy transition (Halstead et al., 2019).

Moreover, our selection of these seven innovations reflects fairly new innovations or phenomena that have grabbed public and policy attention and been reported widely, making them more amenable to studying visions. While electric vehicles and automated vehicles are both linked to energy use in transport, the former is generally envisioned as an energy efficiency innovation and the latter as a more radical shift in mobility patterns. While most low-carbon visions of the future rely on technology, we thought it important to consider a non-technological case study. Fossil fuel divestment is thus a social innovation envisioning new forms of democracy and economics.

Given that we had the benefit of drawing from research as part of a five-year research center, we were able to draw on a rich collection of empirical material spread across multiple projects. We were able to analyze transcripts of original semi-structured research interviews (related to fission, shale gas, divestment), the results of extensive document or media content analysis (related to automated vehicles, smart meters, hydrogen), and reviews of scenario analysis (related to battery electric vehicles), as summarized in Table 2. We frame this type of analysis a ‘qualitative meta-analysis’. From this original material, we identify a collective 38 distinct visions that we then analyze and discuss in terms of their common visions, cues, narratives, and ideographs.

## Results: The discursive dynamics of seven low-carbon innovations

The seven innovations and 38 visions gave rise to a rich mosaic of cues, ideographs, and discursive struggles, or contested visions. Here, we briefly outline each vision in turn, with summary data (and frequency counts) presented in Table A1 in the Supplementary Material.

### Automated mobility

Our material revealed seven visions for automated mobility: (1) effortless freight, (2) the educated trucker, (3) entrenched automobility, (4) transformers, (5) a perilous distraction, (6) infrastructural overhaul, and (7) mass unemployment. Three of the visions feature the driver, while four do not. Of the three that prioritize the driver, one envisions automation as a boon for drivers, while two do not. The other four visions prioritize industrial and infrastructure concerns.

#### Effortless freight

This vision emphasizes efficiency gains for the mobility of goods or freight. It points to current inefficiencies – relating to the driver (e.g. driving hours restrictions), vehicle use (e.g. unproductive time during breaks), empty loads, and fuel consumption – and how these can be overcome, leading to increased profitability. Our research sources mention that ‘a 400 percent price performance improvement’ is possible, given that robots and artificially intelligent beings can ‘drive more economic efficiency’ and that ‘robots don’t mind going 45 mph’. The unemployment caused by removing human drivers is framed in this vision as an opportunity to reduce costs and increase productivity. Benefits for consumers are also articulated.

#### The educated trucker

This vision centers on the role of the human driver, reconfiguring this role as a highly skilled position akin to a ship’s captain or an airplane’s pilot. This vision focuses on the ongoing importance of human interaction through the freight industry, with drivers acting as customer service agents and representatives of the company on route. It suggests that aspects of the job that require least skill will be automated first, and therefore retain the ‘highly skilled’ components: ‘drivers are … among the primary beneficiaries’ of automation and that there would be ample ‘opportunities for truck drivers to take on higher skilled roles’. This vision is likely to have emerged in response to industry concern about employment.

#### Entrenched automobility

This vision points to the evolution and emergence of automated technologies, and creates a vision of incremental innovation that is both inevitable and gradual, offering a future that is commensurable with the present. This vision notes the adoption of automated technologies – micro automations – in freight vehicles. Source material noted that automation would underpin ‘stepping stones on the same path’ to automobility and ‘piece by piece’ change. Technologies such as lane assist, cruise control and automatic braking are framed as early steps in automation. It therefore unpacks the ‘automated vehicle’ to show the variety of technologies that sit within. This vision reduces the radicalness of the innovation of automated vehicles by suggesting that they are a continuation of the technologies experienced today and therefore the future is not likely to be radically different.

#### Transformers

This vision somewhat contradicts the incrementalism inherent in ‘entrenched automobility’ by signaling the exceptionalism and transformative potential of automated vehicles. It draws parallels from science fiction to depict a future of robotic and technological dominance. It goes further to suggest that such a future is ‘just around the corner’, and it focuses on and highlights scientific and engineering capabilities to enable such transformations, and builds excitement around the innovations as ‘future technologies that are available today’. The vision talks about how ‘science has well and truly caught up with fiction’ and that we will soon see a future that is ‘more Optimus Prime than human’, ‘R2D2–like’, and resembling of ‘a scene from Blade Runner’. Through such stories, this vision draws the future into the present and offers suggestions of radical transformation and new ways of being mobile and moving freight.

#### A perilous distraction

This vision problematizes the future orientation of automated vehicles, and their capacity to respond to current challenges of the mobility system at large, and the freight industry more specifically. It directly clashes with the transformers vision by emphasizing the long timeframes for development of automated vehicles, arguing that current challenges require imminent attention. Rather than waiting for technological innovation, these advocates contest the transformer fantasy and argue for increasing attention to driver recruitment and retention due to low pay and conditions, low profit margins, relative costs of fuel, and regulatory changes. Those advancing this vision note that ‘drivers have never said “I do too much” or that “I’m too distracted”,’ and that ‘the driver recruitment/retention issue is primarily about respect and pay’.

#### Infrastructural overhaul

This vision stresses the system-wide change that is required for automated vehicles to be operational, and questions the focus on technological and vehicle capabilities, especially its cost and resources involved. This vision notes the need for regulatory as well as infrastructural and perceptual changes that would be required for automated vehicles to become widespread. It talks about ‘preparing road infrastructure’ and ‘painting its lane stripes six inches wide, instead of the standard four, and repainting them annually’. This vision notes the high-tech (e.g. ‘smart roads’) and low-tech (e.g. ‘line paint’) that may become part of the system of automated mobility, and signals the failures of the technology where these infrastructures are not compatible. This vision therefore suggests it will take a great deal of effort to make an automated reality vehicle-ready.

#### Mass unemployment

This vision, contrary to that of the ‘educated trucker,’ relates to the deskilling of truck drivers, and the potential for technological unemployment and displacement of freight operators, and wider system workers, due to automated vehicle innovation. The vision is one of a ‘driverless’ freight future, and assumes technological capabilities for deliveries without human interference. This, however, threatens to make trucking jobs ‘obsolete’ resulting in ‘massive labor loss that can’t be blamed on trade or regulation but solely on technology’, in the extreme it could ‘abolish almost all the driving jobs’ and ‘the long-haul driver becomes more akin to cartoon buffoon Homer Simpson’. This vision is concerned with the widespread unemployment of professional drivers and the associated ‘humiliation’ of deskilled workers who remain in highly automated freight industry work.

### Battery electric vehicles

Six visions arose from our material on electric vehicles (EVs): (8) entrenched automobility, (9) the electric society, (10) the decarbonized grid, (11) a revitalized economy, (12) the reluctant and anxious consumer, and (13) technological disappointment. The visions differ substantively in whether they view EVs as reinforcing conventional patterns of automobility or reforming it.

#### Entrenched automobility

In this vision, EVs offer a continuation of private automobility as usual. The need for emissions reduction is met in the short term through improvements in conventional vehicles, and only later through introduction of hybrids and electric vehicles (or other low-emission vehicles). The emphasis is on gradual, step-by-step change. The slow changeover allows incumbent manufacturers to continue making and selling conventional cars while adapting to make new types of cars, minimizing systemic change and eliminating the need for behavioral change beyond buying a different type of car. The vision emphasizes how ‘the future car market will be dominated by offerings from traditional [manufacturers] who will use their knowledge and experience, and their understanding of customer desires, together with economies of scale, to develop cost-effective new models with gradually increasing use of electrification’. ‘[C]onventionally powered petrol and diesel cars will remain with us for a long time yet, and that the lion’s share of emissions reductions in the short to medium term will come from their improvement.’ Importantly, there are no shocks to the system, no disruption. The transport system continues with little change to the high-demand, private-car owned configuration.

#### The electric society

By contrast, in this vision, new market entrants take advantage of opportunities offered by EVs that lead to radical changes. New functionalities, like ICT connectivity, and innovative, lighter and smaller cars, allow for new designs that take off in urban areas first. EVs cause a disruption in the transport system, and the change is not just technological, as the role of the car is redefined in society, with more opportunities for a linked-up, intelligent system. This vision suggests that ‘the exploitation of the full potential of electric cars requires total revision of the automobile concept’ and that ‘the successful introduction in to the market of EVs and PHEVs is not merely an evolution of the existing vehicle market, but a transformation of it’.

#### The decarbonized grid

This vision centers not on the implications EVs have on mobility patterns, but instead on the environment, defined narrowly as reducing greenhouse gas emissions. In this framing, electric vehicle purchase is seen as consumer engagement with climate change. The vision notes how ‘increasing electrification of powertrains is widely regarded as the most likely route to achieving GHG reduction targets for passenger cars’ and that ‘significant growth in the electric vehicle market [is] required to meet future carbon budgets, and to be on the cost-effective path to economy decarbonization’. Part of this vision involves further technical innovation in terms of vehicle-to-grid systems, so that EVs can ‘offer potential to balance the grid, as their charging could be turned on and off, and secondly vehicle-to-grid (or house) could offer temporary storage’.

#### A revitalized economy

This vision focuses more on how EVs will accrue enhanced employment opportunities, economic competitiveness and growth, especially for the United Kingdom (where we conducted our assessment). The UK could become ‘a leader in some areas of the electric vehicle market’ and over the long term could even play a globally ‘strong role in future electric systems’. A shift to EVs would capitalize on ‘comparative advantage’ and ‘create jobs, rebalance the British economy towards manufacturing and exports, and promote sustainable economic growth in the UK’. This vision also stresses the links between transport and economy, as ‘road transport will continue to be a critical component of human mobility and economic growth around the world’. Unlike the other visions, this one is grounded in very precise forecasts, with ‘domestic growth in the production of plug-in electric vehicles to the levels predicted by the Smart Grid Forum could create gross value added of £16.5 billion in the UK by 2030, and £52 billion by 2050’, helping secure 470,000 jobs as well by the 2040s.

#### The reluctant and anxious consumer

In this vision, people themselves stunt the potential of EVs. They could be reluctant, ignorant or anxious, failing to take up the new technology, endangering either the successful transformation of the personal vehicle market or the successful reduction in emissions. People are depicted as not seeing the benefits of EVs, in which case they need to be ‘educated’, with a noted ‘gap between people’s attitudes towards the environment and their actions through their choice of vehicle and the way they drive.’ The vision looks at behavior from a ‘rational actor’ perspective, suggesting that ‘people tend to discount heavily (or not take into account) future cost savings from fuel economy at the time of purchasing a car, even though it would seem to be in their own interests’. Further, a recurring theme within this vision even problematizes users through the irrationality of ‘range anxiety’.

#### Technological disappointment

In this vision, EV technology fails to deliver on its promises. It suggests that ‘electric cars will remain confined to niche markets such as special purpose vehicles and delivery fleets in inner city areas’. This is mostly down to uncertainty over things like vehicle performance, batteries, and charging infrastructure, and the competition with the internal combustion engine. According to this vision, ‘big question marks still remain over how [EVs] will perform after several years in terms of day-to-day wear and battery rundown’ and ‘the future mass-market success of electric vehicles is highly dependent on breakthroughs in this field’. Similarly, the costs of the charging infrastructure necessary to support EVs are seen as financially restrictive, given that ‘[EVs] are not designed with the UK electricity network in mind’. This means continued use of conventional cars in the short and medium term. Some documents suggest that while EVs might fail, cars powered by other low-carbon technologies, such as hydrogen or biofuels, succeed.

### Smart meters

Six visions also arose from our smart meters material: (14) empowered consumers, (15) the low-carbon grid, (16) future smart innovation, (17) costly disaster, (18) the hacked and vulnerable grid, and (19) families in turmoil. The competing visions highlight the discursive struggle over whether smart meters benefit or harm consumers.

#### Empowered consumers

This vision presents smart meters as a pathway towards creating more empowered consumers. This empowerment can come from a variety of means, including more accurate household energy bills, prosuming through micro-generation at home, easier management of energy use, ending of debt related to energy bills, choosing favorable tariffs, easier switching between suppliers and, best of all, cutting energy bills in the home. The vision suggests that smart meters are key to making energy visible in the home and helping householders make changes to their daily energy routines to reduce energy bills and carbon emissions. The link between visibility, awareness and changed routines is described as ‘empowering’ householders, i.e. fundamentally changing how they relate to, engage with and use energy.

#### The low-carbon grid

This vision explicates the carbon and environmental credentials of smart meters. Our source material described how the rollout of smart meters would result in Britain’s energy system becoming low-carbon. Smart meters are considered to be enabler of a smart grid and therefore opening up opportunities for increasing low-carbon electricity such as from wind and solar. The vision also includes the possibility of integrating smart meters with energy storage, and that national benefits could accrue beyond carbon, such as a smarter and more secure grid.

#### Future smart innovation

This vision emphasizes the contributions that smart meter rollout could offer for industry, innovation, and economic competitiveness. A consistent theme was that smart meters enable smarter services and business models that could increase competition and innovation in the energy market, for instance, creating opportunities for innovative services to be developed, such as time of use tariffs that could also make energy more affordable. Smart meters are linked to smart grids and smart cities futures, leading ultimately to visions where they could enable grids that incorporate information and communication technologies to enhance performance to reduce resource consumption and overall costs. This would lead to ‘an alluring vision of the future, in which civic technology such as traffic lights, smart meters for utilities and public transport could all be connected and the feedback the of data online invaluable’.

#### Costly disaster

This vision depicts the smart meter rollout as a ‘costly disaster’. Here, smart meters are envisioned as a publicly funded technological and financial calamity with inconclusive benefits for consumers, and with outdated and faulty technologies being rolled out to nearly every household in the UK. The national program is considered to be highly complex, where costs might be a lot higher than calculated in current impact assessments; it is a ‘disaster waiting to happen’ or ‘over-engineered and mind-blowingly expensive’. This leads to calls from differing actors for the entire program to be halted, altered and/or cancelled. The vision also elaborates on a diverse set of technological difficulties (e.g. lack of interoperability between suppliers’ meters and technological obsolescence).

#### Hacked and vulnerable grid

This vision notes how smart meters could result in a national energy system subject to hacking or vulnerable to criminals. Part of it also emphasized vulnerability as a sort of dystopian ‘Big Brother’ narrative highlighting how smart meters would enable utilities to investigate people’s energy consumption in the home to the point where privacy was invaded. The vision warns how plans to install smart energy meters in every house will leave families vulnerable to ‘hacker attacks’, creating a potential risk to individual homes, municipal buildings and even whole districts. One source even characterized such hacking possibilities as comparable to a ‘nuclear strike’, suggesting malicious hackers would be able to cut off whole national electricity grids. The vision also suggests that ‘we will see Big Brother taking over our homes as power companies get to micro-manage our energy supply and are given complete access to information about how we live’, and that smart meters will lead to a ‘honeypot of data which energy insurance and marketing companies will inevitably be hungry for’.

#### Families in turmoil

This vision relates to smart meters adding stress or tension to family routines, or worse, breaking families apart. It is grounded in personal anecdotes from householders and studies about changes in family routines once energy feedback technologies were installed in the home. One of the family members, usually the father, would become an energy consumption detective, controlling the other family members’ energy routines, for instance, switching off a light in an empty room. One writer mentioned how smart meters provoked their seven-year-old daughter to shout ‘you’re destroying the planet, Daddy’ as he boiled a kettle and used the tumble dryer. Other sources indicated that smart meters enabled partners to ‘become the amusing nag around the house’ or turn ‘children into a kind of eco-police force’.

### Nuclear power

Our data on nuclear power resulted in five distinct visions: (20) economic prosperity, (21) advanced nuclear skills, (22) weapons that end the world, (23) nuclear seagulls and kids, and (24) financial maelstrom. Four of the visions directly relate to the commercialization of nuclear energy, while the focus on the fear of nuclear weapons largely ignores the fact that a country can embrace commercial nuclear power while rejecting nuclear weapons or, alternately, develop nuclear weapons without embracing commercial nuclear power.

#### Economic prosperity

This vision promotes idea that nuclear energy could provide economic prosperity throughout the United Kingdom in what one respondent referred to as ‘under-invested parts of the country’. This financial benefit took several forms, including direct employment at the facility itself and spin-off employment for members of the local community who benefit from increasing (and increasingly affluent) local populations. In this way, the construction and operation of nuclear facilities was seen to directly enable access to more jobs and, in so doing, boost economic prosperity. Indeed, one interviewee went as far as saying: ‘these communities want a nuclear power station, they know this is their lifeline in terms of economic growth and sustaining the economy’.

#### Advanced nuclear skills

This vision suggests that the development and operation of nuclear stations was seen to enable the education of the next generation of nuclear experts both in the local area and further afield. This occurred through specialist training and apprenticeship opportunities that enabled life-long jobs. Given that the facilities in question (Hinkley Point in Somerset and Sellafield in Cumbria) were located in somewhat deprived local areas, this was seen as a lifeline for local residents: ‘there’s an apprentice scheme that they can go on rather than going to work on the land like people would have done 30 or 40 years ago, so they see that as an opportunity’.

#### Weapons that end the world

This vision cautions strongly against the production of nuclear energy and subsequent handling of nuclear waste, because it engendered visions of military linkages (both at home and abroad), militarization and nuclear weapons. It includes fears over facilities both exploding like bombs themselves and providing opportunities for them, muddying the waters between energy and war. Referencing the history of the Sellafield Nuclear Complex, for instance, an interviewee stated that it was seen ‘as both a huge technical hope and a quasi-military establishment, making the atoms for peace and atoms for war distinction quite problematic’. This notion was also linked to the idea of proliferation and military threat: ‘[I]t is the ultimate threat, is it not? It is the ultimate weapon, it is a thing you can threaten your neighbor with in a way that no other weapon can.’

#### Nuclear seagulls and kids

This vision touches upon the fear inherent in radiation and nuclear accident through nuclear seagulls and nuclear kids. It stems from both recorded cases of negligence as well as the potential for future accidents. Indeed, commenting on poor maintenance levels, the vision talks about how nuclear energy is ‘absolutely horrifying’ with ‘open-air ponds, which have nuclear waste in them’ being home to seagulls. The vision notes how human health can be affected in a potentially deadly way as well, noting that ‘we are messing around with things and making things that have the potential to mess with the DNA of everything. I fail to see how we are ever going to contain it and stop it from doing that’. The vision also underscores a loss of trust in some nuclear operators.

#### Financial maelstrom

This vision rests on the idea that nuclear energy is incredibly expensive both because of the costs associated with its construction and megawatt strike price, and because of the long-term nuclear waste legacy. Fears emerged that this prohibitive expense – much of which comes from subsidizing the energy form’s competitiveness – would be passed on to the ‘public purse’ and that UK residents would be paying for ‘the most expensive electricity in the world’. The vision discusses how ‘a substantial amount of overpayment for the contract and that is going to result in loading more costs into UK energy bills’ and that ‘we are spending billions every year on trying to deal with it, and I don’t think we are getting very far’.

### Shale gas

Our material led to the identification of five visions for shale gas in Eastern Europe: (25) empowerment of economic opportunity, (26) enhancer of energy security, (27) driver of decarbonization, (28) environmental blight and (29) energy authoritarianism and exploitation. Two central discursive struggles emerge, one based on the competing visions of environmental implications, and one featuring different economic visions. A third point concerns whether the contrast between enhancing national security through independence comes at the risk of marginalization of local communities.

#### Empowerment of economic opportunity

This vision embraces shale gas as a source of economic welfare and job creation, of revenues for national or local budgets, and on the competitiveness of the manufacturing industry that constitute the core of this frame. As our source material indicated, shale gas could make Eastern European citizens ‘soon be living like in Kuwait’ and could ‘lead to decreasing [energy] prices and higher stability’ for the economy. Shale gas development therefore promises ‘plenty of benefits for economic development, … not only to the oil and gas sector, but the overall industry of the country’, ‘especially for the state and local budgets’.

#### Enhancer of energy security

This vision comes against the backdrop of Eastern Europe’s historical experience of being geographically located between major regional powers, Russia and Germany, and the geopolitical fallout of their rivalry or alliances, notably during the period preceding the Second World War. It is equally informed by Eastern Europe’s experience as a group of satellite states of the Soviet Union for much of the postwar period. Reliable energy supply, notably with a view to reducing dependence on Russian gas imports, therefore forms an integral part of this vision, most prominently in Poland. Respondents note that energy is a ‘foreign policy tool for Russia [and] shale gas opens up the possibility of being more secure from Russia's monopolistic position.’ This vision supposes the advancement of ‘independence in the energy sector’ and of ‘long term security of supply’. Shale gas therefore fosters ‘diversification of primary energy sources’ which is deemed ‘of utmost importance’. Other material focused on promoting shale gas for regional ‘sovereignty’ and underlining ‘shale gas is not only an industry, it’s geopolitics’.

#### Driver of decarbonization

This vision sees unconventional gas as an opportunity to decarbonize Eastern European economies. The broader context here is a coal-intensive heat and power sector in the region, for which natural gas may offer a less CO2-heavy energy source (European Commission, 2011). Natural gas is also viewed as helpful in balancing intermittency problems when enhancing renewable electricity generation (Holz et al., 2013; Neumann and von Hirschhausen, 2015). Against this backdrop, the vision suggests that ‘shale gas [on the basis of] best available technologies could be a transition fuel that could complement the use of renewables’. It depicts shale gas as a ‘bridging fuel for renewables’, a position supported by environmental groups adopting a ‘pragmatic understanding’ of (shale) gas as a way to mitigate emissions within national economies. Others talk about shale gas in the context of more stringent EU climate policies and the viability of it helping Eastern Europe if carbon pricing regimes are established.

#### Environmental blight

This vision sees shale gas as a stain on the environment and a threat to ecosystem services that intertwine with agriculture and economic sustainability. This vision was especially prominent in Bulgaria but resonated across the region, referring to the importance of aquifers for crop production and serving the population with drinking water. The vision includes recognition that potential pollution caused by shale gas extraction therefore entails the risk of depriving parts of the Bulgarian society of their economic base, particularly concerning groundwater safety and food security. Other themes within the vision include things like methane leakage, the global warming potential of natural gas, and the sub-optimality of shale gas compared to other decarbonization options such as energy efficiency or renewable sources of energy.

#### Energy authoritarianism and exploitation

This vision labels shale gas as an attempt to exploit domestic resources for private gains. In this vision, foreign energy companies, rather than being perceived as engines of growth and sources of investment, are portrayed as the cause of unsustainable economic activity: ‘they do not create jobs for the local population [only for] foreign experts’. The vision also suggests that shale gas brings ‘no gains for the local population, only damages and problems’ and ‘when things start to collapse, companies just give up, but environmental problems remain’. A related theme within this vision suggests that shale gas promotes energy authoritarianism because a lack of transparency erodes democratic and participatory governance. This aspect of the vision focuses on how the public have lacked ‘sufficient information’ about associated risks, how ‘concessions were granted without public discussion, and an alleged ‘secrecy’ and ‘haste’ pertaining to related policy decisions, fostering distrust. This distrust comes against the backdrop of negative experiences with extractive industries companies being accused of corruption and human rights abuses.

### Hydrogen

We see five hydrogen visions arising from our material as well: (30) patriotic energy independence, (31) the ubiquitous and clean hydrogen economy, (32) energy democratization, (33) climatic disaster, and (34) costly mistake. Struggles over these visions relate to whether they promote decentralization and disrupt energy markets, or further consolidation and corporate control.

#### Patriotic energy independence

Under this vision, clean, hydrogen powered automobiles and power stations are seen as a way to minimize and even eliminate costly dependence on foreign sources of fuel, all the while contributing to economic growth. Hydrogen improves security of supply, minimizes dependence on imports, and improves diversification. In this future world, ‘Environmental pollution will no longer be a concern. Every nation will have all the energy it needs available within its borders. Personal transportation will be cheaper to operate and easier to maintain. Economic, financial and intellectual resources devoted today to acquiring adequate energy resources and to handling environmental issues will be turned to other productive tasks for the benefit of the people. Life will get better.’ By minimizing and eventually displacing imports of foreign fuels, the hydrogen economy is envisioned as an important tool to reduce national deficits, insulate economies for fuel shocks, and improve economic vitality. At the extreme, hydrogen ‘can satisfy all the needs of human kind and form an energy system that would be permanent and independent of energy sources’. It can be blended with undertones of patriotism as well, given that ‘energy independence through hydrogen is a patriotic duty’.

#### The ubiquitous and clean hydrogen economy

This vision of a future where hydrogen is ubiquitous is depicted as an extrapolation of current trends, because shortages in the supply of conventional fossil fuels will force it, or because hydrogen ‘is the indispensable Kyoto compatible, clean and abundant energy carrier’. Sources articulate that ‘the dominant role of hydrogen in a sustainable energy future is widely accepted’ and that hydrogen ‘will come to the fore within the first half of the twenty-first century’. It is envisioned that hydrogen is instrumental to maintaining current levels of consumption and economic growth in the industrialized and industrializing world. Hydrogen is the ‘forever fuel’ or ‘dream fuel’ because it never runs out, and when used to produce power the only byproducts ‘are pure water and heat’. We are told that hydrogen ‘automatically solves, in principle, the global problem of the greenhouse effect’, that it is ‘the ultimate step in climate stabilization’, that it will ‘contribute to the reduction of energy-linked environmental impacts, including global warming’, and that it ‘could be crucial to the future of mankind and the planet it ever-more-tenuously occupies’.

#### Energy democratization

This vision describes the hydrogen economy as a path towards community empowerment and democratization. This theme envisions hydrogen as promoting more pluralistic, participatory, and community-owned forms of energy production. Some proponents even go so far as to frame the hydrogen economy as a fundamental altering of ecological values, changing the way that humanity conceptualizes its relationship with energy technologies and the environment. A more moderate version of this theme shifts from arguing that hydrogen empowers people to a more nuanced debate over the merits of decentralized energy supply. To proponents of this vision, the key to replacing the current energy infrastructure is decentralization, or more particularly the advent of the ‘energy internet’ or the ‘Worldwide Energy Web’. Taken to its logical extreme, this decentralization of energy production transforms society. It will ‘make possible a vast redistribution of power’ eliminating the ‘centralized, top-down flow of energy, controlled by global oil companies and utilities’. The result is peer-to-peer energy sharing, analogous to file sharing on the internet, forcing energy companies to cooperate ‘or follow the evolutionary path of the dinosaurs’.

#### Climatic disaster

This negative vision suggests that using hydrogen as an energy carrier creates fundamental problems unavoidable by the laws of physics and thermodynamics. Hydrogen is a source of energy only if it can be taken in its pure form and reacted with another chemical, such as oxygen. Natural forces have already oxidized all the hydrogen on earth, with the exception of hydrocarbons, so that none of it is available as usable fuel. The rest has to be ‘made’. Oil refineries use hydrogen to purify fuels, and chemical manufacturers employ it to make ammonia and other compounds. Both industries obtain a vast majority of their hydrogen from high-temperature processing of natural gas and petroleum. The method, however, is inefficient, energy-intensive and highly polluting. The vision highlights the inefficiency of using natural gas as a transitional fuel to make hydrogen, and it warns that we should not base policy on unproven technologies, such as the desire to unlock the ‘mysteries that Nature has long kept hidden’.

#### Costly mistake

This negative vision states that pathways for making hydrogen are exceptionally expensive, and would need to see existing costs for energy and electricity rise significantly in order to be competitive. Large, capital-intensive hydrogen infrastructure would have to be erected, including long-distance pipelines and storage facilities. Hydrogen pipelines would also rely on large amounts of energy to move the gas along the line. Storing hydrogen in its gaseous state requires large, high-pressure cylinders, requiring significant storage space. The vision also questions the safety attributes of hydrogen, given that it is flammable at a much wider range of concentrations than natural gas, and hydrogen flames are barely visible. Even under a best-case scenario in which researchers throw an unlimited amount of money into hydrogen research, the vision warns that commercialization would not likely occur until after 2035. This means hydrogen ‘won’t provide cheap and abundant energy’ and that it is a ‘dismal excuse for comprehensive energy solutions’.

### Fossil fuel divestment

Lastly, four visions of fossil fuel divestment were apparent: (35) the climatic imperative, (36) the carbon bubble, (37) democratic transformation, and (38) fiduciary duty. These visions are particularly interesting because they reflect the ways in which cues resonate with informed audiences. The climatic imperative is similar to other visions in this analysis; the arguments invoke a dystopian future, but the narratives can resonate with a larger range of people. The other three visions assume a more technically skilled or informed audience, turning carbon reserves into investor exposure, arguing for the democratic value of divestment, and exploring the role of fiduciary duty. These cues assuredly resonate with investment personnel and business executives.

#### Climatic imperative

This vision asserts that global warming could cause devastating damage to the environment and society, and that limiting global warming to 2°C requires that the majority of known fossil fuel reserves must be left in the ground. This is a moral argument, seeing averting such damage as a higher imperative than economic gain. People articulating this vision talk about ‘doing something’ about climate change, making statements such as ‘you shouldn't be profiting from wrecking the planet from causing worse climate change’ and ‘if you were to extract all of the existing reserves that we have over the world, then that would be enough to cause catastrophic climate change’.

#### Carbon bubble

This vision focuses on the economic risk posed by overvaluing fossil fuel assets and companies that produce fossil fuels, and how the correction of the ‘carbon bubble’ could have severe implications. Again, research indicates that limiting global warming to 2°C requires that the majority of known of fossil fuel reserves must be left in the ground, potentially leaving reserves and infrastructure as ‘stranded assets’, the fuels ‘unburnable’ within the carbon budget. In this vision, ‘you have people investing in fossil fuel companies because of the high dividend, but once the dividend stops, you’re going to see a huge amount of [capital] exiting of that market’ and ‘if companies are not preparing themselves for a fossil-free world, they're exposing themselves to serious issues when it comes to the future’. Another statement suggested that ‘we’re talking about a systemic existential crisis that’s portfolio-wide and now quite urgent’. There is a sense of inevitability of change in this narrative. Some corporations and groups that divest or reduce fossil fuels in their portfolios cite narrow economic grounds, acknowledging neither ethical nor environmental justifications, nor giving any credit to activists.

#### Democratic transformation

This vision iterates some of the political and justice themes in divestment, and how the act of divestment purges and transforms a political system of the corruption and lobbying power of fossil fuels. It depicts the goal of divestment as reducing the power of fossil fuel companies and their financers over the political system, and taking away their social license to operate. Material representing this vision suggested that ‘you’re creating a legitimacy crisis, if you like, for fossil fuel backers and industries’ and that divestment ‘strips fossil fuel companies, the very companies we identify as being the drivers and profiteers of the climate crisis, really strips them of their social license to operate’. These companies need to be ‘called out’ and ‘delegitimized’. In this vision, the fossil fuel industry is the villain, the finance system is its enabler: ‘fossil fuel companies have been deliberately misleading and slowing down progress on climate change’ and ‘the fossil fuel industry is a highly dysfunctional, sociopathic influence on politics and the regulatory system’. The financial sector is ‘the primary enabler of dysfunctional corporate and market behavior’. Divestment is a heroic act of discrediting the industry and issuing a call to arms.

#### Fiduciary duty

This vision opposes divestment based on the obligation of fund managers to act in the best interest of investors and shareholders, often narrowly interpreted as maximizing (short-term) returns on investment. The argument goes that as long as fossil fuels offer the best returns on investment, fund managers cannot legally divest from them, even when that means ignoring the threat of climate change. It is captured in statements such as: ‘we have a fiduciary duty to make a maximum return on our investment’, ‘if they want a good dividend yield, that is what they get from oil and gas companies at the moment’, and ‘trustees have to concentrate on their fiduciary responsibility, primarily’. It suggests there are no alternatives to fossil fuels in terms of safe, profitable investment and highlights that fossil fuels are currently still necessary. It tends to frame campaigners as not understanding the world of finance, nor appreciating the need for fossil fuels. It notes that there is hardly any fund ‘which looks like a normal stock market fund for charities which has no fossil fuel extraction and refinement activities in it’.

## Rhetorical features: Problems, storylines and discursive struggles

Unveiling 38 visions across seven innovations is an arduous task, and we believe there is novelty in simply identifying such rhetorical diversity (with, again, a full list offered in Table A1 in the Supplementary Material). However, in this section, we critically and comparatively analyze all visions according to the problems they attempt to address, common storylines and characters, and discursive struggles and contestations.

### Problems, functions and ideographs

At their core, many visions are addressing some sort of problem – therefore possessing a functional utility rather than merely a symbolic one. This confirms earlier research suggesting that visions and fantasies are often functional and instrumental, or even utopian, because they fulfill some perceived social need, enabling proponents to capture resources (Geels and Smit, 2000). For example, the development of hydrogen may play a role in the creation of social communities, reflecting a desire for empowerment and democratization under conditions of decentralism and localism. This discursive relationship between problem and solution can serve to broker relationships between relevant social groups and create a dynamic of ‘promise and requirement’ where actors make promissory commitments to the technology, forging a shared agenda that requires action. In this way, the functionality of the vision results in a ‘mandate’ to developers and advocates, what Borup et al. (2006: 290) call ‘the freedom to explore and develop combined with a societal obligation to deliver in the end’.

When looking at solutions (or non-solutions, on some visions) to problems, the visions are fairly vague, which likely enhances their rhetorical appeal. As a result, the problem being addressed can become a symbolic cue. To the extent that the cues resonate with audiences, individuals can fill those gaps according to their interpretations of the visions. The dynamism between specific problems and solutions implies a ‘rhetorical selectivity’ (Peterson, 1997: 12) that obscures meaningful barriers or problems with a low-carbon innovation (Sovacool and Ramana, 2015). For example, electric vehicles are portrayed as a technical solution to the narrowly defined problem of carbon emissions in a way that overlooks all other problems caused by automobility (except perhaps local air pollution); this vision relies on defining sustainability as an emissions problem only, obscuring the challenge of decarbonizing a grid needing the additional capacity to charge EVs. Thus, new technologies become evaluated primarily as solutions to existing problems (as defined by the technology’s advocates).

We see similar dimensions, cues, functions, problems and ideographs across the 38 visions depicted earlier. As Table 3 indicates, some visions center on technological or scientific problems or ideographs such as progress, innovation, scientific exploration or technical development. Some center on socio-economic dimensions such as poverty, jobs, and growth. Some focus on environmental concerns such as sustainability, futurity, stewardship or energy efficiency. Some involve security in various forms – national security, human security, safety, individual privacy, and terrorism. Some relate to politics – liberty, democracy, empowerment, decentralization and independence become known. Indeed, in some (electric vehicles, smart meters), there is even a shifting of responsibility from the technology and its manufacturers to users, framing uptake – and consumers – as a problem.

**Table 3.** Recurring dimensions, problems and ideographs in low-carbon visions.

Dimension	Examples	Illustrative problems (and possible cues)	Ideographs
Technological and scientific	Effortless freight (automated vehicles), transformers (automated vehicles), a perilous distraction (automated vehicles), the electric society (electric vehicles), technological disappointment (electric vehicles), future smart innovation (smart meters), costly disaster (smart meters), costly mistake (hydrogen)	Traffic congestion, road accidents, phlegmatic innovation patterns, inferior performance, prohibitive costs	Progress and innovation, efficiency, resistance
Socioeconomic	The educated trucker (automated vehicles), entrenched automobility (automated vehicles and electric vehicles), infrastructural overhaul (automated vehicles), mass unemployment (automated vehicles), a revitalized economy (electric vehicles), economic prosperity (nuclear), advanced nuclear skills (nuclear), financial maelstrom (nuclear), empowerment of economic opportunity (shale gas), the carbon bubble (divestment)	Lack of skills, unemployment, globalization and protectionism, economic recession, unstable financial markets	Profit and economic growth, employment, education
Environmental	The decarbonized grid (electric vehicles), the low-carbon grid (smart meters), nuclear seagulls and kids (nuclear), driver of decarbonization (shale gas), environmental blight (shale gas), the ubiquitous and clean hydrogen economy (hydrogen), climatic disaster (hydrogen), climatic imperative (divestment)	Climate change, degradation of water quality, air pollution, radioactive waste	Environmental sustainability, safety
Security	The reluctant and anxious consumer (electric vehicles), hacked and vulnerable grid (smart meters), families in turmoil (smart meters), weapons that end the world (nuclear), enhancer of energy security (shale gas)	Resource scarcity, household vulnerability, military strength, arms races, geopolitical instability	Safety, privacy, security, resistance
Political	Empowered consumers (smart meters), energy authoritarianism and exploitation (shale gas), patriotic energy independence (hydrogen), energy democratization (hydrogen), democratic transformation (divestment), fiduciary duty (divestment)	Illiberal values, populism, energy dependence, corruption	Liberty and autonomy, duty and responsibility, resistance

### Storylines, characters and plots

Although less explicit and detailed, our visions differ in terms of their stories and narratives, plotlines, agency and characters. Sometimes, agents play active and conscious roles; at other times, they play passive or even subconscious/unknowing roles. Some may be essential to securing a particular type of future, acting as a ‘star’ or ‘lead actor’; others may be important but not critical (acting as ‘extras’ or ‘costars’) (Brown et al., 2000). Others may touch upon themes such as heroism or horror (Janda and Topouzi, 2015).

Propp (1968) offers a useful framework for analyzing the plots of stories, building on an analysis of Russian folktales. All of the folktales Propp examined have common themes such as morphemes (analyzable pieces) and narratemes (narrative units). He identifies five common elements across those stories:

- predatory functions of the donor (the term Propp uses), or common functions of the dramatis personae (the characters), such as being unmarried, going to war, striking it rich, and so on;
- conjunctive elements, or common rhetorical techniques to amplify the message, such as characters raising their voice or the sudden announcement of misfortune to enhance drama;
- motivations, or the reasoning, goals, aims, and strategies of the characters;
- forms of appearance, or how the characters entered and exited (e.g., flying on the back of a dragon, or arriving unexpectedly by chance);
- attributive elements, or material artifacts and accessories that move the plot along (e.g., a weapon, a cave, a witches’ hut, a castle).

Given that most of our low-carbon visions were relatively short and technical, they do not fully meet Propp’s framework or contain all of these elements. However, our visions do conform to two of Propp’s findings. Our visions are *paired*, they almost always have the presence of good versus evil in the form of heroes (kings, soldiers, unmarried bachelors, and eagles for Propp) pitted against villains (a dragon, a devil, bandits, a witch, or a stepmother for Propp). And our visions are full of *attributive elements*, especially material technologies and socio-technical systems, which can influence the trajectory of the story in active and passive ways.

**Figure 1.**
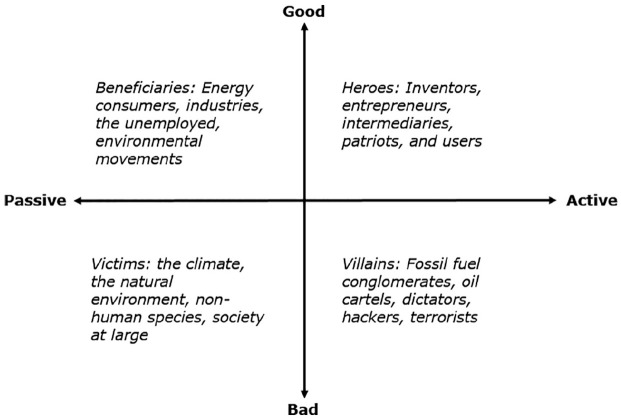
Heroes, villains, beneficiaries, and victims in low-carbon visions.

Numerous types of actors and agents come up throughout our visions, fitting the typology of pairing (heroes and villains) and attributive elements (passive vs. active) described by Propp (1968) (see Figure 1). Within our material, these included:

- the product or artifact: the technology or sociotechnical system with limitless potential;
- the happy user or consumer: the likely or intended adopter of the particular technology or service (or conversely the reluctant, anxious, or irrational consumer that rejects the innovation);
- the ally or intermediary: the critical stakeholder or champion whose support is needed for the innovation or technology to succeed;
- the prime adversary: the single person, stakeholder group, institution, or policy regime that must be overcome or eliminated;
- the daunting challenge: the massive degree of social or technical transformation needed to ensure the vision occurs;
- the evil villain: the malicious and deceptive provocateur set to destroy the world who must be stopped at all costs;
- the inhuman opponent: faceless threats such as climate change, poverty, or human insecurity that must be thwarted.

Almost every vision is paired, it can be tied in some way to a *villain* or *adversary*. Under the theme of progress (for automated vehicles, electric vehicles, and hydrogen in particular) are those who, often through ignorance and self-centeredness, doubt the legitimacy of a foregone and beneficial energy transition. The *villains* in the themes of independence or autarky (smart meters, hydrogen, divestment) are fossil fuel companies and financial actors who support them, oil suppliers, energy companies and cartels such as OPEC that desire to raise energy prices or invest in fossil fuels. Moreover, in some instances, visions involve more specific actors such as hackers and terrorists behind a vulnerable grid (smart meters), or the corporate stewards adhering to notions of fiduciary duty (divestment). The villains, adversaries and challenges even become conflated in the themes of effortless freight (automated vehicles), the electric society (electric vehicles), future smart innovation (smart meters), patriotic energy independence (hydrogen), or the ubiquitous and clean hydrogen economy (hydrogen) – those that seek to waste energy, subvert innovation, and/or and select inefficient technologies. More generally, environmentalists across almost all of the visions see big industrial emitters of greenhouse gases as culprits threatening the vitality of the climate, whereas industrial stakeholders may see environmentalists as villains seeking to constrain growth and place limits on industrialization.

Some visions center on *inhuman opponents* such as climate change, degraded habitats, or disruptions to ecosystem services (or *passive victims* such as the climate or environment). These visions include the decarbonized grid (electric vehicles), the low-carbon grid (smart meters), nuclear seagulls and kids (nuclear power), driver of decarbonization (shale gas), environmental blight (shale gas), the ubiquitous and clean hydrogen economy (hydrogen), climatic disaster (hydrogen), and climatic imperative (divestment). Other visions revolve around institutions, politicians, energy conglomerates, and other firms as the main *adversaries*, such as empowered consumers (smart meters), energy authoritarianism and exploitation (shale gas), patriotic energy independence (hydrogen), energy democratization (hydrogen), and democratic transformation (divestment).

Many visions have *passive agents* or *passive beneficiaries*. These are people or groups that are either ignored or presumed to play their part in a simplistic way. For example, electric vehicle visions tend to portray users/consumers as passive actors, whose sole role in the transition is to buy a different type of vehicle, rather than act as partners, agents of change, or knowledge providers; behavior change beyond vehicle choice is mostly ignored.

The resonance of these stories, characters, villains and challenges reminds us that the publics and audiences subscribing to a particular vision will develop or reuse code words, phrases, slogans and themes. To give just a few examples, we see ‘robots’ repeatedly mentioned in visions of automated automobility, ‘Big Brother’ and ‘spies’ mentioned frequently in smart meter visions, and shale gas constantly heralded as a ‘bridge’ to a low-carbon future. These cues can trigger previously shared fantasies, may refer to a geographical or imaginary place or the name of a persona, and they may arouse a range of other emotions. This act of cuing a narrative enables groups of people to come to a cognitive convergence about that part of their common experience. These visions can advance discussions at pragmatic levels, but once cataclysmic depictions of problems are articulated, the narrative forms tend to lead advocates to advance utopian fantasies for solutions or dystopian fantasies to visualize causes. The fluidity of problems, functions, and ideographs serves as a reminder that each of the visions associated with particular energy systems may be aimed at particular audiences or publics. Bryant (1953) notes that rhetoric performs ‘the function of adjusting ideas to people and of people to ideas’. Put simply, effective advocates manipulate their messages to resonate with their audiences, but they also use messages to help move audiences to be receptive to ideas.

### Discursive struggles and contestations

Each of our visions contains internal discursive struggles or contestations. For visions about automated vehicles, uncertainty remains over the extent that automation will facilitate (or destroy) jobs and skills, as well as over whether they will transform or simply reinforce traditional forms of mobility. Similar contestations arise over whether electric vehicles will condone or challenge conventional forms of automobility, as well as whether they will require behavioral adjustment (adapting the consumer to the car) or further innovation (adapting the car to consumer needs). Smart meters exhibit struggles over where the financial and carbon savings accrue (among consumers, suppliers, or society as a whole?) and to the extent that consumer data is protected or merely sold to interested companies and government agencies. Nuclear visions are contested over the timing and extent of projected costs and benefits as well as where (geographically) external costs are distributed. Shale gas visions contest whether it will complement incumbent energy providers or disrupt them, as well as whether it is a bridge to a low-carbon energy system or a costly detour. Hydrogen visions also contest whether it will empower communities to challenge the dominance of conventional energy companies or merely reinforce their innovativeness and competitiveness, as well as its cleanliness in terms of how hydrogen is manufactured. Visions about divestment remain contested over whether moral and environmental imperatives ought to outweigh financial ones, and whether the fossil fuel industry is a partner or a predator when it comes to a low-carbon society. Furthermore, visions also take contested views over particular ideographs, with as one example some (electric vehicles) seeing safety as inherently *not* disrupting the system, whereas others (divestment, hydrogen) see disruption as key to a safer future.

Thus, despite their utopian undertones as solving compelling problems, the imagined futures across our sample are also contested and contradictory. In such cases, contradictions can be strategic. As Berkout (2006: 305) suggests, ‘a degree of flexibility over the interpretation of a vision can widen its relevance to greater numbers of actors’. Some contradiction can relate to the manufactured ambiguity or flexibility of most fantasies: They need to be broad enough to enroll actors but vague enough to withstand criticism. In other cases, such contradictions can reflect internal denial and cognitive dissonance among articulators of the vision or its audience. When looking at the early history of innovations such as electricity, x-rays and the telegraph, Simon (2005) suggests that the introduction of any potentially transforming technology creates a tension between desirable changes in day-to-day life and the anxiety that follows any step into the unknown. For example, automated vehicles reflect to some an appealing way of enhancing the knowledge and skills base of human drivers (comforting), yet to others they offer a mechanisms by which human drivers may be entirely redundant and thus locked out of millions of jobs (increasing anxiety).

More broadly, some visions promote low-carbon innovation on the grounds that it would radically reorient society to hold more ecologically sustainable values – essentially transforming the energy system – whereas others promote visions that enable us to continue business as usual, preserving and extending the energy system. We will even return to this point below when discussing the incremental versus radical nature of the 38 visions. As Tenner (2006: 64) writes, however, this tension reflects a deeper social dilemma: ‘the choice is between a material and artistic culture that reflects and even anticipates change and one that cushions the spiritual shocks of change.’ Marshall (2016) adds that some visions and fantasies act as psycho-social defense mechanisms, intended to assuage political discomfort or social anxiety. Here, the contradictory nature of a vision is not a weakness or an unintended byproduct, but its intended strength or purpose.

### Rhetorical effectiveness and resolution

A final implication of this analysis features the rhetorical implications of the competing visions or their discursive struggles, and, in doing so, identifies routes advocates can consider to enhance their persuasive appeals. The discussion of divestment provides a valuable example. It is difficult to imagine that someone would prioritize short term economic gain over substantial and realistic degradation of the planet. What then, are the obstacles to achieving the solution advanced in the vision? Part of it is still convincing some people that the threats of anthropomorphic climate change are real, and part may be to convince others that we can do something about it. However, a persuasive argument that may work is to target current laws concerning the fiduciary duty of companies to maximize profits for investors. Striking such laws seems implausible, yet modifying them to exclude practices that actively harm the planet may be possible. The specifics of such a proposal are well beyond the scope of this paper, but an important element this analysis can provide is revealing the most important conflicts in visions, thus setting up calls to look for new arguments that tackle particular discursive struggles which arise from the existing conflicting visions.

Similarly, rather than feature a need for ‘more education’ about the benefits of EVs as a response to the reluctant and anxious consumer vision, this analysis might suggest that people are not always rationale, and that suggests contemplating different persuasive strategies. For example, extending ranges, identifying adoption with national or planetary goals, or finding collaborative approaches so that individuals do not feel that they are making significant personal sacrifices while achieving little for the planet since relatively few of their peers are buying EVs, could all provide alternatives to efforts to provide more education. In any of these competing visions, finding the points of contestation is a significant contribution that can provide an initial step toward finding resolutions.

## Typologies: Valence, temporality and radicalism

Visions differ not only in their rhetorical features; they vary meaningfully in dichotomies or typologies in terms of the valence (utopian vs. dystopian), temporality (proximal vs. distant), and radicalism (incremental vs. transformative).

### Valence (utopian vs. dystopian)

One fundamental way visions differ – already alluded to in the discussion of problems, functions, and ideographs – is their *valence*. Some visions are utopian, and tied to positive emotions such as hope, excitement, happiness, and even love. Others are dystopian, and tied to negative emotions such as fear, despair, boredom, and even hatred.

For example, some of our visions frame low-carbon innovation as a harbinger of a utopian democratic social order (hydrogen), a nirvana for technical innovation and business development (smart meters), a platform for automobility ubiquity (automated mobility, electric vehicles), or a pathway towards environmental sustainability and decarbonization (shale gas, fossil fuel divestment, others) (see Table 4). These starkly contrast with negative visions of radiation, fear and death (nuclear power), businesses declaring bankruptcy (fossil fuel divestment), terrorists and hackers launching new sophisticated attacks on grids (smart meters), and consumers held hostage to the whims of unsentimental corporate firms (shale gas).

**Table 4.** Utopian and dystopian valence of 38 low-carbon visions.

Innovation	Visions	
	Positive and utopian	Negative and dystopian
Automated vehicles	Effortless freight, the educated trucker, transformers	Entrenched automobility, a perilous distraction, infrastructural overhaul, mass unemployment
Electric vehicles	Entrenched automobility, the electric society, the decarbonized grid, a revitalized economy	The anxious and reluctant consumer, technological disappointment
Smart meters	Empowered consumers, the low-carbon grid, future smart innovation	Costly disaster, hacked and vulnerable grid, families in turmoil
Nuclear power	Economic prosperity, advanced nuclear skills	Weapons that end the world, nuclear seagulls and kids, financial maelstrom
Shale gas	Empowerment of economic opportunity, enhancer of energy security, driver of decarbonization	Environmental blight, energy authoritarianism and exploitation
Hydrogen	Patriotic energy independence, the ubiquitous and clean hydrogen economy, energy democratization	Climatic disaster, costly mistake
Fossil fuel divestment	Climatic imperative, democratic transformation	Fiduciary duty, the carbon bubble

Furthermore, some visions directly refute or challenge each other. For automated vehicles, the vision of the educated trucker is the opposite of one of mass unemployment. For electric vehicles, all of the positive visions would be negated by a technological disappointment; for smart meters, future smart innovation is literally offset by a costly disaster. For nuclear power, economic prosperity is the antithesis of financial maelstrom, and weapons to end the world would trump anything positive at all arising from advanced nuclear skills (and, arguably, anything else for that matter). For shale gas, environmental blight is the literal opposite of the driver of decarbonization vision, the gains from economic opportunity are offset by energy authoritarianism. For hydrogen, the theme of energy democracy sees communities taking back control over production and use, whereas a theme of patriotism instead sees control shifted to companies and national economic competitiveness enhanced. For divestment, a climatic imperative could come at the massive expense of a carbon bubble crippling economies. This tension in valence suggests a dynamic dialectic in that the positive visions are often defined only in relation to the negative ones (they avoid them), and vice versa. In other cases, tensions in valence can be a way of furthering the plot – of enhancing the performative effect of a vision’s climax, resolution, or failure to reach a resolution (Deuten and Rip, 2000).

### Temporality (proximal vs. distant)

Visions can differ in their *temporality*, some are proximal (depicted to occur within a few weeks to a few years’ time) whereas others are distant (far into the future, often a decade or even a century away).

Recognizing that many visions fall into more intermediate areas between proximal and distant, Table 5 sketches of the temporal dimensions of the 38 visions. Automated vehicles could bring improvements in efficiency and effort and skills development or costly infrastructural investments within the coming decade, whereas other visions such as transformers or mass unemployment would be more distant. EVs, by contrast, could entrench automobility or be hobbled by anxious adopters in the near term, or decarbonize grids and revitalize the UK economy in the long-term. Smart meters can empower consumers or lead to hacked grids now, whereas smart innovation and truly low-carbon grids come later. For nuclear power, one must suffer the risk of weapons that end the world and nuclear seagulls and kids to achieve the more distant prosperously revitalized economy or enduring nuclear skills. Shale gas sees environmental impacts and local economic effects occur in the near-term, but potential impacts on energy security or the carbon intensity of whole economies in the longer-term. Similarly, hydrogen’s risk of exacerbating climate change or sinking investment in the wrong pathway come before one could ever hope to achieve true energy independence, a hydrogen economy, or democratization. For fossil fuel divestment, all of the visions are proximal, given the urgency of tackling climate change, although their consequences and implications are for the more distant future.

**Table 5.** Proximal and distant temporality of 38 low-carbon visions.

Innovation	Visions	
	Proximal (within the next decade)	Distant (within at least a decade)
Automated vehicles	Effortless freight, the educated trucker, a perilous distraction	Entrenched automobility, Transformers, mass unemployment, infrastructural overhaul
Electric vehicles	Entrenched automobility, the reluctant and anxious consumer	The electric society, the decarbonized grid, a revitalized economy, technological disappointment
Smart meters	Empowered consumers, families in turmoil, hacked and vulnerable grid	Future smart innovation, low-carbon grid, costly disaster
Nuclear power	Weapons that end the world, nuclear seagulls and kids	Economic prosperity, advanced nuclear skills, financial maelstrom
Shale gas	Empowerment of economic opportunity, energy authoritarianism, environmental blight	Enhancer of environmental security, driver of decarbonization
Hydrogen	Climatic disaster, costly mistake	Patriotic energy independence, the ubiquitous and clean hydrogen economy, energy democratization
Fossil fuel divestment	Fiduciary duty, climatic imperative, the carbon bubble, democratic transformation	

An implicit element of visions with differing timescales and immediacy (or temporal remoteness) is that some benefits may be pitted against each other in temporal terms. As Brown et al. (2000: 4) write, future expectations ‘may run in parallel with and contest each other, occupying different time-frames and carrying different interests’. Some visions concern benefits and risks, such as economic development or water contamination, that occur now, while others, such as eventually halting climate change or transformations of social or economic structure, will occur well into the future. They also occur at different scales: things like employment, land, air and human health impacts tend to be localized, whereas progressive growth, the elimination of poverty or energy dependence, solving climate change, or cascading patterns of innovation are national or even international. Such complexity plays a forceful role in making visions contested – something touched upon above – and it also implies that the particular array of costs and benefits will play out differently according to each vision but mediated by temporality and place. The future will likely hold even more diverse and divergent pathways than these.

### Radicalism (incremental vs. transformative)

A final way visions meaningfully differ is in terms of whether the *scope of sociotechnical change* brought about by the vision is incremental, pragmatic or conventional, or instead radical, disruptive and transformative (Michael, 2000). Incremental visions essentially see the future as similar to the present, taking current fundamental or foundational conditions as the basis of foresight. These visions may even seek to protect, extend or entrench business as usual. This contrasts with transformative visions that are more progressive, disruptive, substantive or ends-oriented, in which society may differ in fundamental ways from how it exists now.

We see such dynamics at play within our 38 visions, summarized in Table 6. Automated vehicles could either incrementally improve the efficiency of freight or the skillset of drivers, or lead to the wholesale overhauling of transport infrastructure and rampant disruption of labor markets. EVs can either entrench or entirely reform mobility. Smart meters can either empower consumers or place them at the mercy of errant hackers and terrorists. Nuclear power can either enhance national prosperity or lead to the apocalypse of all nations. Shale gas can protect incumbent firms or lead to entire transformations of ecosystems and climatic damage. Hydrogen can either lead to business as usual (freed from environmental constraints) or revolutionize global energy and political systems. Divestment can similarly remind investors of the merits (and legal necessity) of pursuing business as usual or also see the entire collapse of national economies or (corrupt) political systems.

**Table 6.** Incremental and transformative dimensions of 38 low-carbon visions.

Innovation	Visions	
	Incremental or protective	Transformative or disruptive
Automated vehicles	Effortless freight, the educated trucker, entrenched automobility, a perilous distraction	Transformers, infrastructural overhaul, mass unemployment
Electric vehicles	Entrenched automobility, the reluctant and anxious consumer, technological disappointment	The electric society, the decarbonized grid, a revitalized economy
Smart meters	Empowered consumers, costly disaster, families in turmoil	Future smart innovation, low-carbon grid, hacked and vulnerable grid
Nuclear power	Economic prosperity, advanced nuclear skills	Weapons that end the world, nuclear seagulls and kids, financial maelstrom
Shale gas	Empowerment of economic opportunity, enhancer of energy security, energy authoritarianism	Driver of decarbonization, environmental blight
Hydrogen	Patriotic energy independence, climatic disaster, costly mistake	Ubiquitous and clean hydrogen economy, energy democratization
Fossil fuel divestment	Fiduciary duty	Climatic imperative, the carbon bubble, democratic transformation

## Conclusion

Our evidence revealed 38 visions and 14 ideographs circulating across a mere seven low-carbon innovations dealing with mobility or energy. Based on this evidence, we advance five synthetic conclusions.

First, many visions are contextually specific to the innovation being examined, such as the educated trucker (automated mobility), the reluctant and anxious consumer (electric vehicles), families in turmoil (smart meters), nuclear seagulls and kids (nuclear power), energy authoritarianism (shale gas), ubiquitous hydrogen economy (hydrogen), and the carbon bubble (fossil fuel divestment). Others seemed eerily similar to each other or are fairly generic across our innovations, such as entrenched automobility (electric vehicles, automated mobility), decarbonization (electric vehicles, smart meters, shale gas, divestment), democracy (hydrogen, divestment), and various visions of economic growth or stability (electric vehicles, nuclear power, shale gas).

Second, despite their varying specificity and generalizability, the 38 visions involve a diverse set of storylines and cues, addressing different problems across different dimensions. A priori, we can imagine problems in: scientific, technological, social, cultural, economic, environmental, security, political, moral, epistemic, aesthetic, religious, health and logical dimensions. Obviously, some of these dimensions are more salient to future energy visions than others. Also, the popularity and recurrence of many visions across each innovation suggests that the broader low-carbon future remains an open-ended idea, as well as one subject to mass appeal, capable of sustaining the public imagination. Within these visions, there do remain apparent ‘master narratives’ and ‘cues’ with broad consensus, reflected in high frequency counts. The most frequent visions were effortless freight (63% across our automated vehicles evidence), entrenched automobility (94% of electric vehicle evidence), empowered consumers (64% of smart meters), nuclear kids and seagulls (61% of nuclear power evidence), financial maelstrom (61% of nuclear power), economic empowerment for shale gas (49% of evidence), patriotic energy independence (65% of hydrogen), and the carbon bubble (92% of divestment material). In some cases, a single dominant vision could reflect a growing consensus among powerful incumbents over the most desirable future, such as the entrenched automobility vision. In contrast, there are areas where competing visions do not result in ‘high’ frequency counts, which may suggest that competing visions are still contested, cues ineffective and fantasies unformed. In the various visions of automated trucking, the vision of effortless freight was very high (63%), but whether that was good for drivers was not settled. The educated trucker vision was moderate (35%), while two negative visions had low representations. The perilous distraction vision was found in 12% of the material, and the mass unemployment vision was found in 18% of the material.

Third, and perhaps obviously, such visions are complex and dynamic. Low-carbon innovations are depicted in the narratives as capable of achieving things as diverse as eroding family values (smart meters), eliminating poverty (hydrogen), reasserting individual or local autonomy (automated vehicles, electric vehicles, shale gas, nuclear power), or leading to the end of the world (nuclear power) or attacks akin to nuclear strikes (smart meters). Such visions weave together and develop stories that intersect energy production and consumption with diverse topics across domains and scales, and also rely on a variety of distinct symbolic cues. Yet others are about foreclosing options or ‘colonizing the future’ (Middleton, 2015) by closing down storylines: optimistic and inspirational visions can ignore or erase likely challenges; despondent and even apocalyptic visions can discount potential advantages in their attempts to motivate people to reject and actively resist a dystopian future. Some imagined futures are incremental, whereas others predict sweeping, radical changes, underscoring how seemingly incremental changes to technology (electric motor, grid interconnectivity, fuel cells) can lead to visionary storylines.

Moreover, the centrality of low-carbon, sustainability and/or energy efficiency is highly variable within the broader narratives of the different innovations but also within the various visions. These themes may be used or coopted by particular agendas to gain legitimacy and/or support – for instance, niche actors in vehicle automation are making sweeping and often highly generalized claims of benefits which, to date, are largely unsupported, context specific and contingent on a wider suite of assumptions about the energy system.

Fourth, as predicted, ideographs – a rhetorical manifestation of some ideology or recurring ideal – are recurrent across our visions. Visions at a narrow level about a cleaner type of natural gas, a safer or more ‘British’ reactor, a more widely dispersed fuel cell or smart meter or vehicle become about much more than that. They borrow from and connect with deeper ideographs of progress, environmental sustainability, liberty and autonomy, privacy, duty, and security (to name a few). Interestingly, visions can even deploy the same ideograph in starkly different ways, i.e., some ideographs of liberty and autonomy focus on ‘freedom to’ – to explore, innovate or build skills – whereas others emphasize ‘freedom from’ – from unemployment, insecurity, poverty or a degraded environment.

In this way, visions may fulfil a general social need for fantasy, and as such will likely continue to exist even as the specific innovations behind a low-carbon society change and evolve. On the other hand, some visions are created and used by powerful actors, pursuing their own agendas, and utilizing a social need for visions to further their own end, such as limiting the spread of automation or electrification to conventional vehicles, or constraining the effects of smart meters or divestment. Indeed, sustainability in a majority of visions is seen narrowly as reducing greenhouse gas emissions, and that this can and has to be done via technological change – hinting that it does not necessitate other more difficult changes to behavior, lifestyle or social structure; in other contexts, the (environmental) sustainability ideograph can mean much more, considering nuclear waste and destroyed habitats for example. This is an example of the ‘flexibility’ of ideographs, and how they can be used differently in different contexts and by different actors. Visions can be used to open some futures and close others, and to narrowly redefine or even shut out some narratives.

Fifth, and last, the fantasy themes undergirding these 38 visions imply that the choices made by analysts, politicians, users, scientists and other stakeholders about low-carbon innovation are not always purposively rational. The prevalence of these visions strongly suggests that current discussions and broader narratives about energy technology and policy are seamlessly intertwined with compelling (and exciting and at times frightening) fantasies. The decisions we each make about energy systems transcend economic self-interest, logic, and rationality and involve elements as diffuse as mass fantasy, individual optimism, dramatic storylines, symbolic cues, communal hope, heroes and villains, contradictions, business ambition, national pride and fear of uncertainty.

Because it fulfills these deeper needs, the provocative force of fantasy can positively stimulate and shape investment decisions, research trends and sociotechnical pathways that come to reject undesirable options and embrace desirable ones. Alternately, by distorting from the rational towards our emotions and dreams, visions can enable powerful actors to hide serious problems and encourage incomplete solutions, clouding our judgment. Energy fantasies and exaggerated rhetoric can become particularly hazardous if they blind us to the realities of new energy sources, and more mundane opportunities for energy efficiencies or reductions in energy consumption, by promising a golden tomorrow only by ignoring the stark and growing problems of today. But they can also motivate us to imagine and hope for possibilities that empower us to escape or perhaps transcend those very limitations. We need to both drive for particular, purposeful and transformative visions that resonate with a diverse set of stakeholders, but also acknowledge their discursive struggles and contestations to ensure such visions are more socially appropriate and legitimate if they begin to become a reality.

## Supplemental Material

TableA1-Submitted-BKS – Supplemental material for Imagining sustainable energy and mobility transitions: Valence, temporality, and radicalism in 38 visions of a low-carbon future Click here for additional data file. Supplemental material, TableA1-Submitted-BKS for Imagining sustainable energy and mobility transitions: Valence, temporality, and radicalism in 38 visions of a low-carbon future by Benjamin K Sovacool, Noam Bergman, Debbie Hopkins, Kirsten EH Jenkins, Sabine Hielscher, Andres Goldthau and Brent Brossmann in Social Studies of Science
