# Supplementary material for: Imagining sustainable energy and mobility transitions: Valence, temporality, and radicalism in 38 visions of a low-carbon future
Source: Soc Stud Sci. 2020 May 6;50(4):642–79. doi: 10.1177/0306312720915283 (PMC7399846; doi:10.1177/0306312720915283)
Supplement: TableA1-Submitted-BKS – Supplemental material for Imagining sustainable energy and mobility transitions: Valence, temporality, and radicalism in 38 visions of a low-carbon future [file TableA1-Submitted-BKS.pdf]

Table A1: Summary of low-carbon visions, cues, discursive struggles, and ideographs

a. **Automated vehicles**

No.	Vision	Frequency (n=107)	Description	Cues	Discursive Struggles	Ideograph(s)
1	Effortless freight	Very high (n=67/107)	Automation of the driving task will make the movement of goods, and the industry more efficient, benefitting industry (increased profitability) and consumers (improved service). Efficiency will be gained through reduced fuel consumption, improved safety, reducing/eliminating costs of human drivers, and restrictions of driver hour regulations.	Boosting efficiency; reducing emissions; significant fuel savings; the payoff is large; kind of a no brainer; dramatically cheaper operation costs; eliminate the driver; enhancing truck utilization.	1). Automation allows operating efficiency for freight businesses 2). Consumers will benefit by way of improved services 3). Efficiencies will result in environmental sustainability by way of reduced fuel consumption.	Efficiency, progress and innovation, profit and economic growth
2	The educated trucker	Moderate (n=37/107)	Automated technologies will make the work of truck and van driving more desirable, removing the boring and mundane tasks, and instead allowing drivers to take on other, more interesting and challenging tasks. Drivers will oversee the driving task, becoming transport managers, and taking on greater administrative and customer service tasks.	More pay for less work; more like the captain of a ship; conditions probably will be better; truck drivers stand to benefit; drivers remain necessary; higher level of sophistication and specialisation; lead to higher driver pay; make the job more appealing.	1). The current 'low-skilled' role of the freight driver will be replaced by a 'high-skilled' position that will be more highly paid and appreciated 2). Driving is a task that professional drivers find tedious.	Employment, education, liberty and autonomy

3	Entrenched automobility	Moderate (n=26/107)	The emergence of automated technologies is part of an evolutionary process that builds upon existing in-vehicle technologies. It is not, therefore, a radically different future (that should be a source of concern) but an inevitable future, with increasingly automated vehicles.	It is not a question of how… but when; semi-autonomous features already are being incorporated into today's trucks; organically grow into an autonomous vehicle; trucks will evolve component by component, system by system; an evolutionary process; a continuation; inevitably become the norm.	1). Automated vehicles are part of a trajectory towards increasingly automated vehicles.<br>2). Full automation is inevitable.<br>3). Innovation is not radical but a natural progression.	Safety
4	Transformers	Moderate (n=33/107)	A rapidly evolving Sci-Fi future, that is more than fantasy, and turning dreams into reality. The pace of change is rapid, and the industry and wider governance context must prepare themselves for this vastly different future reality.	Robots even have permeated the trucking industry; sooner rather than later; a future guided by computers; next generation transport; no longer a sci-fi pipedream; fast moving; visionary; closer than we think; transformation.	1). Automated vehicles are a 'sci-fi dream come true'.<br>2). Technological wonder of automated vehicle innovation.<br>3). Transformational technology.	Progress and innovation
5	A perilous distraction	Low (n=13/107)	Automated technologies are framed as a distant fantasy, that focus too much on the future, and fail to respond to pressing current problems. The technology is not relevant to the requirements of the goods mobility industry.	Driver welfare; it doesn't necessary offer a solution to a lot of near term issues motor carriers now face; How does a driverless vehicle load and unload itself?	1). Focus on the future can neglect attention to the present.<br>2). Current focus overlooks industry challenges (e.g. low profit margins, low	Environmental sustainability, resistance

					pay, recruitment of drivers).	
6	Infrastructural overhaul	Low (n=15/107)	The technologies are misaligned with current urban form, transport and freight industry infrastructures, policy and regulation. They therefore require more fundamental changes to the built environment to become compatible. Moreover, human-driver tasks (e.g. un/loading)	The need to adjust the highway infrastructure to better support driverless trucks; 45-mph trucks will likely need their own lane; legal framework and regulations; road markings; smart highways.	1). Vehicles require broad ranging system changes to be fully operational.<br>2). Improvement of infrastructure, and development of specific policy and regulation is required.	Progress and innovation, safety
7	Mass unemployment	Low (n=19/107)	Technological unemployment will result from automated innovation, whereby the skill and responsibility of the truck driver is removed and passed to automated technologies. The adoption of technologies will reduce the need for truck drivers, and reduce the level of skill required for those roles that continue. Support-industry jobs (e.g. truck stops) would likely reduce too.	Long-term implications for truckers' jobs or roadside businesses like motels and truck stops are also hazy; potentially a job killer; humiliation; a massive displacement of jobs.	1). Technological unemployment will have direct (e.g. drivers) and indirect (e.g. truck stop café staff) impacts.<br>2). Those jobs that remain will be 'deskilled', and further lower the perception of the industry.	Employment, profit and economic growth

b. Electric vehicles

**No.**	**Vision**	**Frequency (n=16)**	**Description**	**Cues**	**Discursive Struggles**	**Ideograph(s)**

8	Entrenched Automobility	Very high (n=15/16)	Automobility continues. Emissions met by incremental improvements. Slow change avoids disruption and allows incumbents to adapt. Link of car manufacture and road transport to economy crucial.	Petrol and diesel cars will be with us for a long time yet; gradually tighter emissions targets; low hanging fruit; transport will emit less while playing vital role in economic growth; buying greener car is addressing climate change.	1). Consumer behavior centered on choice of car.<br>2). Uncertainties (uptake, technology, policy) to be addressed by government	Profit and economic growth, environmental sustainability, safety
9	The electric society	Very low (n=3/16)	Automobility remains, but there are changes. EVs offer the Promise of new technology, toppling some incumbents and changing the nature of car.	EVs are here to stay; full potential requires revision of car concept; new business models, leasing; EVs offer economic promise. EVs are inevitable.	1). Revolutionizing the car, while somehow maintaining automobility.	Progress and innovation, profit and economic growth, environmental sustainability
10	The decarbonized grid	High (n=12/16)	EVs decarbonize and clean passenger transport, lead to storage of renewables	Smart-metering; ICT connectivity; EVs balance grid, offer storage; change starts in the cities;	1). Sources of electricity supply, especially disagreements over nuclear power, fossil fuels (shale gas) and centralized renewables such as hydro	Progress and innovation, environmental sustainability
11	A revitalized economy	Low (n=6/16)	EVs will bring jobs, competitiveness, and growth	UK could be leader in EV production	1). Whether such jobs are in new or conventional industries, whether they are within the UK or overseas	Progress and innovation, profit and economic growth, employment

12	The reluctant and anxious consumer	High (n=11/16)	The success of EVs is held back by people, whose behavior (i.e. car choice) displays ignorance, anxiety or irrationality. Responsibility for EV uptake is shifted from the industry to the consumer. People's lack of acceptance is a barrier to be overcome.	Environmental concern doesn't translate to buying EVs; people discount future savings from EVs; public needs education; consumers have irrational range anxiety; people have technological bias; awareness and acceptance of EVs is low.	1). People 'should' behave as rational actors and buy EVS, but they are not.	Progress and innovation, education
13	Technological disappointment	Medium (n=8/16)	Technology isn't delivering, or there are large uncertainties about cost and timing, or because a 'breakthrough' is needed. In the short and medium term, ICEVs are therefore needed.	Limited range, long recharging time of EVs; uncertainties over cost and range mean mass uptake could be delayed; EV future requires breakthrough in battery technology; EVs are destined to remain a niche.	1). Belief in technological progress is in tension with casting doubt on EV technology.	Progress and innovation, resistance

c. **Smart meters**

No.	Vision	Frequency (n=205)	Description	Cues	Discursive struggles	Ideograph(s)
14	Empowered consumers	Very high (n=131/205)	Smart meters will facilitate consumers to manage and control their energy use/bills through more transparent and accurate bill reading, easier switching between suppliers,	More control over energy usage; better position to bring… bills down; prosuming enabled; take advantage of off-peak deals; accurate bills; switch suppliers more quickly;	1) Savings on energy bills being made (at what point and how high)<br>2) Technologies enable	Liberty and autonomy, profit and economic growth

			choosing flexible tariffs, and prosumption.	end to shocking utility bills; newly empowered consumers	social/financial possibilities (such as switching between suppliers)	
15	The low-carbon grid	Moderate (n=48/205)	Smart meters will promote/ enable the decarbonization of electricity and gas.	Cutting carbon emissions; low-carbon energy future; green revolution; combating climate change	1) cleanliness of electricity supply	Environmental sustainability
16	Future smart innovation	Low (n=14/205)	Smart meters will enhance industrial strategy and economic competiveness.	Boost to competition and innovation in the energy market; more exciting new innovations; new smart home products	1) Geographic proximity of innovations versus spillover beyond the UK	Progress and innovation, profit and economic growth
17	Costly disaster	High (n=105/205)	The smart meter rollout will be a publicly funded technological and financial disaster with inconclusive benefits and outdated and faulty technology	Could cost far more; becoming a costly disaster; government IT disaster; embarrassing and costly failure; uncertain benefits; white elephant; the most complex in the world; danger of speedy obsolesce, replacing old meter is a nightmare; do not work in several types of buildings; benefits that will not justify the costs	1) Cost-effectiveness of the rollout<br>2) Transparency and validity of impact assessments of the rollout<br>3) Substantiality of technological promises	Progress and innovation, profit and economic growth, resistance
18	Hacked and vulnerable grid	Moderate (n=65/205)	Smart meters will erode privacy protections: companies gaining information about people's lifestyles and potentially controlling home appliances;	The 'spy' in the home; able to 'snoop' on people's lives, invasion of privacy; feel even more captive; permanent window on their private life; lack of trust in suppliers;	1) Data being misused<br>2) Acceptability of automated and/or remote control of home appliances	Privacy, security, resistance

			Smart meters can facilitate criminal theft of data or sabotage of the electricity or gas network	'honeypot' of data; Big-Brother-ish, Vulnerable to hacking and manipulation; national cybersecurity vulnerability; foreign computer hackers; hostile act against Britain's critical national infrastructure; leaving the national grid crippled	3) Cyber security risks<br>4) Capacity to remotely cut off networked system	
19	Families in turmoil	Low (n=9/205)	Smart meters will disrupt family routines and can lead to the policing of activity by children, partners and parents	You're destroying the planet; I had to keep nagging my husband; a kind of eco-police force		Safety

d. Nuclear power

**No.**	**Vision**	**Frequency (n=36)**	**Description**	**Cues**	**Discursive struggles**	**Ideograph(s)**
20	Economic prosperity	High (n=18/36)	The construction of nuclear facilities will facilitate surrounding communities to access more jobs through direct and secondary employment (e.g. hotels), enabling boosts in economic prosperity.	Lifeline; primary employer; want the jobs; investment wages highest outside London; bringing jobs and skills; economic growth and sustaining the economy.	1) Direct jobs being provided in local communities (what kind and for whom)<br>2) Indirect benefits stemming from hotel, restaurant and	Progress and economic development, employment

					manufacturing businesses<br>3) Replacement opportunities for dying industries	
21	Advanced nuclear skills	Moderate (n=12/36)	The development of nuclear facilities provides opportunities to educate the next generation of UK nuclear experts and in the local area, provides specialist training and apprenticeship opportunities for practical, life-long jobs.	Youth enter into nuclear industry; apprentice opportunities; STEM subjects at school; impact on local deprivation	1) Establishment of local colleges and school partnerships<br>2) Apprenticeships and work experience<br>3) Training of young experts for a next generation of UK experts	Progress and innovation
22	Weapons that end the world	Moderate (n=13/36)	The production and of nuclear energy and handling of nuclear waste is inherently linked to the military, militarization and nuclear weapons.	Nuclear weapons proliferation; weaponry; it is a thing you can threaten your neighbor with in a way that no other weapon can; militarization; dropped loads of bombs; atoms for peace and atoms for war distinction quite problematic	1) Nuclear production linked with plutonium production<br>2) Nuclear waste linked to submarines and bombs<br>3) International links to military operations e.g. Middle East	Security, progress and innovation, resistance

23	Nuclear seagulls and kids	Very high (n=22/36)	Due to negligence and potential accidents, nuclear energy presents the risk of severe radioactive contamination for both the wider environment and its human inhabitants.	Deadly; potential to mess up the DNA of everything; breath-taking incompetence; chucking waste into swimming pools without even knowing how much you were throwing or where it came from; a disaster waiting to happen; passively safe; have seagulls in them; going to explode like a bomb; contaminated; leaking	1) Facilities not being maintained appropriately<br>2) Potential for accidents including leaks and explosions<br>3) What are the risks for the environment and humans living there<br>4) Trust in operators	Environmental sustainability, safety
24	Financial maelstrom	Very high (n=22/36)	Nuclear energy is incredibly expensive, both in terms of facility construction and megawatt price, and because of the long-term waste legacy.	Public purse; paying for the most expensive electricity in the world; spending billions; loading more costs into UK energy bills; surcharge or levy; increase prevalence of fuel poverty; cash strapped; affordability; very little is free.	1) Strike price per megawatt for new nuclear is very high<br>2) Costs being passed to consumer through energy bills and levies<br>3) New nuclear reliant on subsidies<br>4) Extremely expensive nuclear waste legacy and burden	Economic development and growth

e. Shale gas fracking

No.	Vision	Frequency (n=74)	Description	Cues	Discursive struggles	Ideograph(s)
25	Empowerment of economic opportunity	High (n=36/74)	Shale gas will endow countries and communities with jobs, economic development, tax revenue and in some situations a reindustrialization of the economy	Economic welfare; national competitiveness; job creation, lower prices	1) Extent to which it complements or disrupts existing incumbents	Progress and innovation, profit and economic growth, employment, security
26	Enhancer of energy security	Moderate (n=28/74)	Shale gas will enable countries to liberate themselves from dependence on energy imports (particularly from Russia) and enhance national security	Energy independence; diversification strategy; geopolitical stability; sovereignty	1) Degree to which energy independence trades off with other dominant visions	Security
27	Driver of decarbonisation	Low (n=19/74)	Shale gas will assist countries in their national climate change policies and hedge against decarbonisation policy risks	Decarbonisation; transition fuel; bridging fuel; carbon pricing	1) Environmental monitoring	Environmental sustainability, profit and economic growth
28	Environmental blight	Moderate (n=23/74)	Shale gas threats water quality and availability, risks chemical pollution, and can accelerate species loss and the destruction of habitats	Groundwater safety; toxic pollution; habitat destruction; agricultural contamination	1) Environmental safety and human security	Environmental sustainability, profit and economic growth, employment, safety
29	Energy authoritarianism and exploitation	Moderate (n=22/74)	Shale gas decision-making is opaque, can concentrate political power, and marginalize local communities;	Distrust; foreign control; exclusion and exploitation; antidemocratic; democratic and participatory governance	1) Legitimacy of public consultations and institutional	Profit and economic growth, liberty and autonomy,

			shale gas production merely transfers wealth and revenue out of domestic economies to foreign actors		procedures underpinning licensing<br>2) Transparency of policy process, decision making and revenue flows	security, inevitability, authoritarianism/ exploitation

f. Hydrogen

No.	Vision	Frequency (n=62)	Description	Cues	Discursive struggles	Ideograph(s)
30	Patriotic energy independence	Very high (n=40/74)	Hydrogen offers a domestic and secure source of local energy	Security of supply; enhancing energy security; energy independence; fuel shocks; economic vitality	1) Extent to which national improvements to security trade-off with other countries and regions	Profit and economic growth, security, environmental sustainability
31	The ubiquitous and clean hydrogen economy	High (n=37/74)	Hydrogen can meet growing demand for energy in a limitless and environmentally benign matter	Moving away from fossil fuels; Kyoto-friendly energy; forever fuel; dream fuel; climate stabilization; limitless consumption	1) Synergies with other low-carbon sources of energy (wind, solar)<br>2) Degree to which continued economic and energy consumption growth can be truly limitless	Profit and economic growth, progress and innovation, environmental sustainability
32	Energy democratization	Moderate (n=16/74)	Hydrogen engenders a decentralized and more	Local energy; energy democracy; decentralization;	1) Tensions between	Liberty and autonomy,

			localized world	community energy; autarky; energy internet; worldwide energy web	democratic community choices and aggregate effects on energy systems	employment
33	Climatic disaster	Moderate (n=20/74)	Hydrogen is an energy intensive and wasteful process that is a folly to pursue for environmental reasons	Hydrogen manufacturing; energy penalty; inefficiency	1) Sources of energy used to manufacture hydrogen	Environmental sustainability, progress and innovation
34	Costly mistake	High (n=31/74)	Hydrogen is a costly source of energy that would require trillions of dollars of investments to catalyze on a large-scale	Cost effectiveness; storage; pipelines; distribution; hydrogen leaks	1) State of technological readiness	Safety, profit and economic growth, progress and innovation, resistance

g. **Divestment of fossil fuels**

No.	Vision	Frequency (n=12)	Description	Cues	Discursive struggles	Ideograph(s)
35	Climatic imperative	Moderate (n=5/12)	Climate change could cause catastrophic damage to the environment and society. The moral imperative of abandoning fossil fuels outweighs economics.	Leave it in the ground; unburnable carbon; catastrophic climate change ; profit from wrecking the planet	1) moral imperatives of environmental and social harm outweigh financial arguments	Environmental sustainability, safety, security
36	The carbon bubble	Very high (n=11/12)	There is economic risk in overvaluing fossil fuel assets and companies. Portfolios heavy in fossil fuels leave investors exposed. As the economy	Fossil fuels no longer safe investment ; carbon bubble ; there will be stranded assets; companies not preparing are exposing themselves to risk;	1) Risk of stranded assets and revaluing fossil fuel companies not sufficiently	Profit and economic growth, national security

			transitions, there will be stranded assets.		appreciated by markets	
37	Democratic transformation	High (n=8/12)	Divestment aims to reduce the power of fossil fuels and their financers over the political system by taking away their social license. Investments in fossil fuels companies are unethical. The fossil fuel industry is the villain.	Delegitimize the fossil fuel industry ; strip them of their social license ; we don't invest in arms companies, gambling, tobacco, and pornography. ; We've finally identified that fossil fuel companies are the enemy	1) The real goal of divestment is not financially hurting the fossil fuel industry, but revoking its social license and its legitimacy.	Liberty and autonomy
38	Fiduciary duty	High (n=10/12)	Fund managers cannot divest from fossil fuels as they are legally obligated to offer the best returns on investment.	Maximize return on investment; good dividend yield; no eligible funds ... that are fossil free; at the moment fossil fuels are essential; can't screen investments on these grounds; Who are these people to come and tell us what we should do	1) Incorporating climate risk (et al.) into fiduciary duty 2) Investments should align with a charity's mission	Profit and economic growth, duty and responsibility, resistance

Source: Authors.
